# Spatially multiplexed dark-field microspectrophotometry for nanoplasmonics

**DOI:** 10.1038/srep22836

**Published:** 2016-03-08

**Authors:** V. Pini, P. M. Kosaka, J. J. Ruz, O. Malvar, M. Encinar, J. Tamayo, M. Calleja

**Affiliations:** 1 IMM-Instituto de Microelectrónica de Madrid (CNM-CSIC), Isaac Newton 8, PTM, E-28760, Tres Cantos, Madrid, Spain

## Abstract

Monitoring the effect of the substrate on the local surface plasmon resonance (LSPR) of metallic nanoparticles is key for deepening our understanding of light-matter interactions at the nanoscale. This coupling gives rise to shifts of the LSPR as well as changes in the scattering pattern shape. The problem requires of high-throughput techniques that present both high spatial and spectral resolution. We present here a technique, referred to as Spatially Multiplexed Micro-Spectrophotometry (SMMS), able to perform polarization-resolved spectral and spatial analysis of the scattered light over large surface areas. The SMMS technique provides three orders of magnitude faster spectroscopic analysis than conventional dark-field microspectrophotometry, with the capability for mapping the spatial distribution of the scattered light intensity with lateral resolution of 40 nm over surface areas of 0.02 mm^2^. We show polarization-resolved dark-field spectral analysis of hundreds of gold nanoparticles deposited on a silicon surface. The technique allows determining the effect of the substrate on the LSPR of single nanoparticles and dimers and their scattering patterns. This is applied for rapid discrimination and counting of monomers and dimers of nanoparticles. In addition, the diameter of individual nanoparticles can be rapidly assessed with 1 nm accuracy.

Metal nanostructures exhibit localized surface plasmon resonances (LSPR) due to collective coherent electron oscillations confined at the nanoscale^1^,^2^,^3^. Different LSPR modes can be supported by a single metal nanostructure, which gives rise to peaks in the extinction and scattering spectra. The intensity, wavelength and spectral width of such peaks are determined by the size, shape, and material of the nanoparticle, as well as the optical properties of the surrounding environment. The capability to tune the plasmon resonances of metallic nanoparticles and the characterization of such responses is at the core of research in nanoplasmonics^4^,^5^,^6^,^7^,^8^,^9^ and it is essential for the development of plasmonic particle-based therapies^6^, plasmonic detection of biomolecules^10^,^11^,^12^,^13^,^14^,^15^,^16^,^17^,^18^, photo-catalysts^19^, plasmonic rulers^20^,^21^, nanoantennas^22^,^23^,^24^,^25^ or surface enhanced Raman spectroscopy^26^. The optical response of a plasmonic nanoparticle is sensitively modified through the interactions with the supporting surface or with the presence of neighbouring nanoparticles at nanometer scale distances. These effects must be characterized and understood for the rational design of plasmonic devices. Importantly, these plasmonic coupling effects allow further tailoring of the plasmonic properties and the appearance of interesting new phenomena^25^,^27^,^28^,^29^,^30^,^31^ such as Fano-like resonances^29^,^32^,^33^,^34^, that may be harnessed for enhancement of the figures of merit of plasmonic devices.

Dark-field microspectrophotometry, based on either nanoparticle scattering^32^,^35^,^36^ or extinction^37^,^38^,^39^, is a fundamental technique for the characterization of plasmonic devices and metallic nanoparticles. A key figure of merit of the technique is the capability to perform spectral analysis of individual nano-objects. The spectral characterization of single scatterers by dark-field microspectrometry is particularly useful to achieve quantitative comparison to theory^40^, to derive information about the morphology or composition of materials^41^,^42^,^43^,^44^,^45^ and to optimize the performance of devices based on plasmonic nanoparticles^46^,^47^. In most of current microspectrophotometers, a white light beam is tightly focused onto the sample surface through a microscope objective and the scattered light coming from the sample is collected and coupled to an optical fiber or to an entrance aperture of an optical spectrometer. Still, this technique suffers of important limitations in throughput, speed and spatial resolution. First, the spatial resolution is limited by the size of the collection spot, typically of few μm and not better than 500 nm in the most optimized instrumentation^48^. Second, the precise positioning of nanoparticles onto the small collection area is commonly achieved by the use of piezoelectric translation stages^48^. Thus, the spatial mapping of spectral properties of large numbers of nanoparticles on extended areas, or even the full spatial characterization of the emission from a single nanoparticle requires the movement of the sample and the stitching of the resulting images. This procedure implies that, for a fixed collection area, the time measurement scales quadratically with the ratio between the size of the studied region and the collection area diameter determining the spatial resolution. Thus, high-throughput characterization is compromised by the spatial resolution. In addition, stitching of multiple images is prone to errors upon image reconstruction. Due to the low throughput and poor spatial resolution, the characterization of large numbers of nanoparticles, either isolated or forming assemblies, with actual micro-spectrophotometers is a non-trivial task. Fast characterization of the plasmonic properties of large numbers of nanoparticles with high spatial resolution remains a demand for the advancement in the design, characterization and application of devices based on plasmonic nanoparticles^27^. High spatial resolution is also demanding for the study of the spatial emission of plasmonic nanoparticles and the entanglement of the responses of the diverse modes supported by the same nanoparticle. These effects play an important role in applications that require the out-coupling of the plasmon resonances into far-field signals for sensing and processing.

In this work, we present a technique, referred to as Spatially Multiplexed Micro-Spectrophotometry (SMMS), able to perform polarization-resolved far-field spectral and spatial analysis of the scattered light over large surface areas. The high spatial and spectral resolutions of the SMMS technique allow fast and accurate optical characterization of hundreds of single and dimer nanoparticles.

## Results and Discussion

### SMMS technique

The spectral characterization of the scattered light by a sample surface requires the acquisition of a tridimensional data set commonly known as spectral cube. The 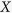 and 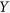 coordinates of the spectral cube represent the sample surface, while the third coordinate corresponds to the light wavelength, λ. In state-of-the-art micro-spectrophotometers, the full spectral analysis of a single point on the sample surface is acquired in a one-shot measurement, while the spatial mapping is performed by sequentially scanning point-by-point the sample surface. Micro-spectrophotometers are thus multiplexed in the spectral coordinate λ and sequential in the spatial coordinates 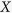 and 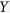. The SMMS technique described here performs one shot measurement of the scattered light by the sample surface at each fixed wavelength, and sweeps sequentially over the desired range of wavelengths. Thus, the SMMS technique is multiplexed in the spatial coordinates, whereas it is sequential in the spectral analysis.

**Figure 1:**
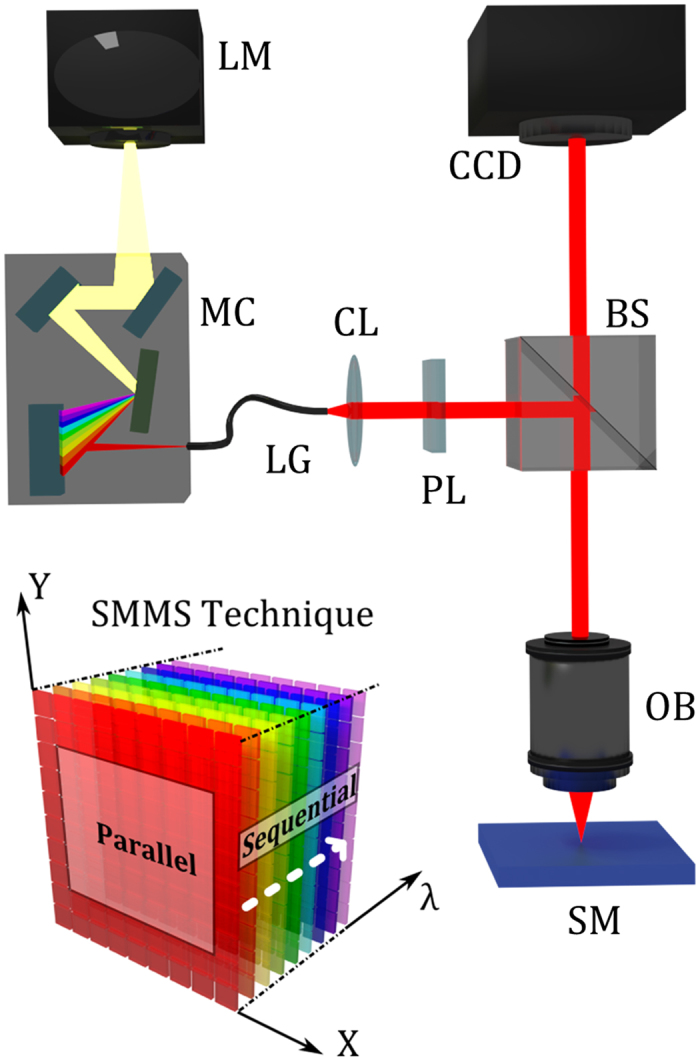
Working principle and practical realization of the SMMS technique in reflection mode configuration. The white light from a lamp (LM) is directed to a motorized monochromator (MC) that disperses the light into its constituent wavelengths. A narrow band of the dispersed spectrum passing from the exit slit of the monochromator is directed to a collimator (CL) and a linear polarizer (PL) through a light guide (LG). Thus, monochromatic light is directed and focused to the sample (SM) by a beam splitter (BS) and a dark-field objective (OB). A high resolution CCD camera placed at the image plane of the experimental setup collects, for each illumination wavelength, λ, the scattered light coming from the full sample surface area. The SMMS technique can be employed also as a standard optical microscope by making use of the zero-order diffraction of the monochromator grating. The left bottom inset is a schematic drawing of the SMMS working principle; the measurements are acquired in parallel (multiplexed) for the spatial coordinates X and Y, and sequentially along the spectral coordinate λ.

Figure 1 shows a scheme of the SMMS experimental setup configuration in the reflection mode. The white light from a lamp (LM) is directed to a monochromator (MC) that disperses the light into its constituent wavelengths. A narrow band of the dispersed spectrum is then coupled to a light guide (LG) and then collimated and focused to the sample surface (SM) through a collimator (CL) and a dark-field objective (OB), respectively. A CCD camera placed at the image plane of the experimental setup collects, for each illumination wavelength λ, the scattered light coming from the sample. A linear polarizer (PL) has been placed in the illumination path to control the polarization of the incident light. More technical details about the practical realization of the SMMS setup can be found in Methods Section.

The measurement of large sample areas using the SMMS technique, typically ranging from several hundreds of μm^2^ up to few mm^2^, is much faster than the standard micro-spectrophotometric techniques, because the use of control stages for the movement of the sample is not necessary. Moreover, the absence of an optical fiber along the detection arm ensures better robustness and easier maintenance to the SMMS technique.

### Polarization-resolved Spectral Analysis of Plasmonic Nanoparticles

**Figure 2:**
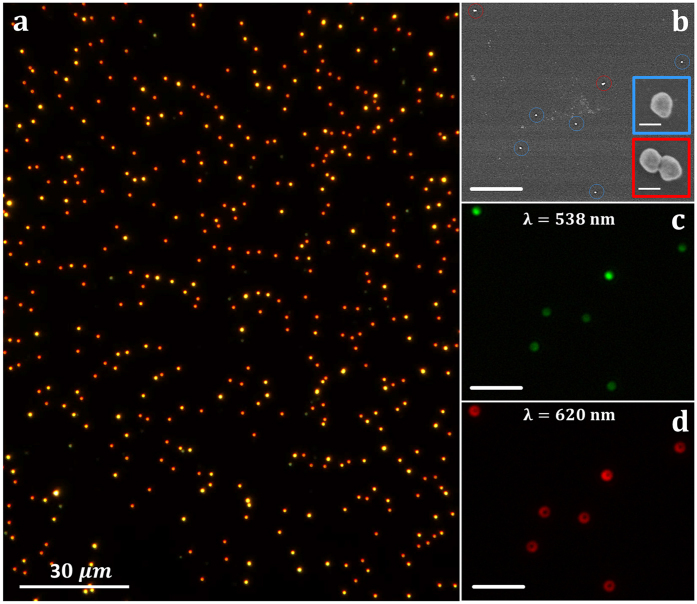
Optical and SEM images of gold nanoparticles deposited onto a silicon substrate. (**a**) Optical dark-field image of 100 nm diameter gold nanoparticles deposited onto a silicon substrate. The sample surface is illuminated here with a broad light spectrum by using the zero-order diffraction of the monochromator grating. (**b**) SEM image of a zoomed area of 16 μm × 19 μm, where single nanoparticles and dimers are respectively identified by blue and red circles. The scale bar shown in the figure corresponds to 4 μm. The insets show SEM images of one monomer and one dimer nanoparticle selected from those shown in (**b**); white bar scales correspond to 100 nm. (**c,d**) Dark-field optical images of the same surface area shown in (**b**), obtained at two different wavelengths; 538 nm (**c**) and 620 nm (**d**). The scale bars correspond to 4 μm.

We apply here the SMMS technique for the dark-field spectral analysis of hundreds of 100 nm diameter gold nanoparticles deposited onto a silicon substrate (see Fig. 2). We prepared the sample to have a low nanoparticle density, of less than 1 nanoparticle per 10 μm^2^, to ensure the presence of individual nanoparticles within a diffraction limited area; more technical details about sample preparation can be found in Methods Section. The measurements were carried out by using a 100× dark-field objective (LU Plan Fluor 100×, Nikon, numerical aperture N.A. 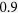)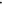 Although the magnification of the objective is high, the detection area is still large, of 163 μm × 125 μm (0.02 mm^2^), thus ensuring we can achieve spectral characterization of light scattered from hundreds of individual nanoparticles in 2 minutes.

A dark-field image of gold nanoparticles deposited on silicon is shown in Fig. 2a. The image was obtained by illuminating the sample with a broad light spectrum, condition that is achieved by employing the zero-order diffraction of the monochromator grating. The sample under study contains mainly a mixture of single and dimer nanoparticles, as confirmed by SEM imaging, as shown in Fig. 2b. Single and dimer nanoparticles are marked by blue and red circles, respectively. In our experimental conditions trimers, tetramers and larger agglomerates are detected as saturated scattering signal and were discarded from the analysis. Figure 2c,d show the far-field scattering of the same SEM surface area of Fig. 2b obtained at two different wavelengths, 538 nm and 620 nm. Single and dimer nanoparticles present two different scattering patterns depending on the wavelength selected: i) a typical Airy pattern with green light illumination (538 nm) and ii) a doughnut-shape emission with red light (620 nm). The observed phenomenology is related to the presence of the dielectric substrate that provides a mechanism for symmetry-breaking of plasmon resonances^32^,^49^,^50^,^51^. While a spherical nanoparticle in an isotropic environment exhibits three degenerate dipolar resonances, the reduced symmetry induced by the presence of the dielectric results in the splitting of these modes into two resonance peaks, corresponding to two plasmonic dipoles oscillating parallel (S-mode) and perpendicular (P-mode) to the surface substrate. The energy splitting of the two modes is induced by their interaction with the underneath substrate and the difference in the spatial distribution of the far-field scattering emission is produced by the different orientation of the dipole modes with respect to the surface substrate. Moreover, since the optical response of both the S-mode and P-mode have a strong polarization dependence, their scattering efficiency can be selectively attenuated or enhanced by changing the polarization state of the illuminating source^52^.

**Figure 3:**
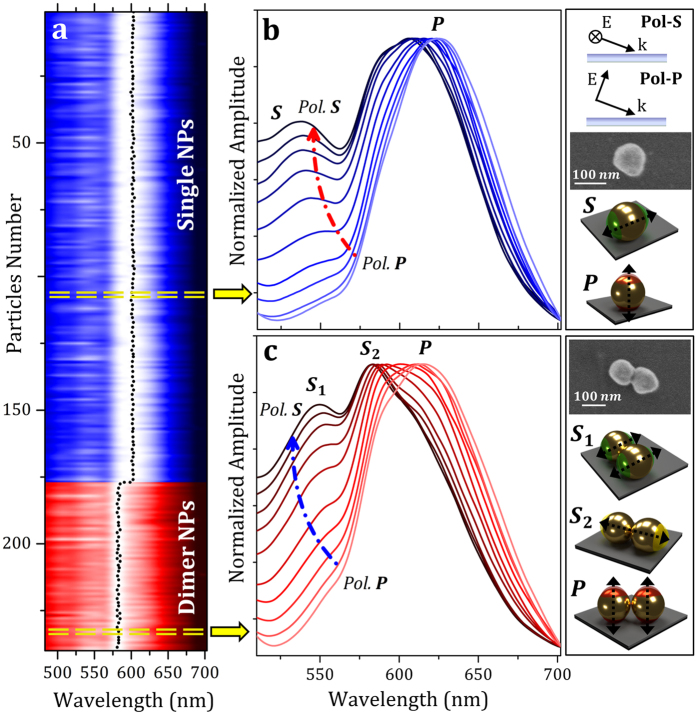
Polarization-Resolved Spectral Analysis of Single and Dimer Nanoparticles. (**a**) Color contour plot of normalized scattering spectra of 177 single (blue color) and 64 dimer (red color) nanoparticles obtained for unpolarized illumination on an imaging area of 0.02 mm^2^. The black dotted lines represent the spectral maximum of each analyzed nanoparticle. (**b**,**c**) Normalized scattering spectra of a monomer (**b**) and a dimer (**c**) obtained at different polarization of the incident light. Insets on the right side show a schematic drawing of the Pol-S and Pol-P illumination source configurations, SEM images of the single and dimer nanoparticles analyzed and a schematic drawing of the dipole plasmon modes observed in the spectra.

Normalized scattering spectra of 241 gold nanoparticles obtained with unpolarized illumination are shown in the color contour plot of Fig. 3a; blue color corresponds to single nanoparticles, while red color belongs to dimers. More technical details about raw data normalization and data analysis can be found in Methods Section. The entire spectral characterization in the visible spectral range (from 480 nm to 700 nm with steps of 1 nm) was obtained by fixing for each selected wavelength an acquisition time of 300 ms; the overall characterization was performed in less than two minutes. Taking a look to the whole nanoparticle ensemble, it is clear how single and dimer nanoparticles have very different spectral fingerprint. As highlighted by the black dotted line shown in Fig. 3a, single nanoparticles have a scattering maximum around 600 nm while dimers more efficiently scatter light close to 580 nm. This spectral behavior is caused by the influence of the high refractive index silicon substrate. In order to analyze in detail the spectral features of single and dimer nanoparticles, polarization-resolved spectral analysis was performed by modifying the polarization state of the illumination light.

The polarization-resolved spectra of representative single and dimer nanoparticles are shown in Fig. 3b,c, respectively. The p-polarized and s-polarized illumination correspond to the electric field direction being parallel and perpendicular to the plane of incidence, respectively (see the inset on the top right). The SEM images in Fig. 3b,c correspond to the nanoparticles under analysis. Polarization-resolved scattering spectra performed on a single nanoparticle (Fig. 3b) show two resonant peaks related to a low energy P mode, around 620 nm, and to a high energy S mode that exhibits its maximum close to 540 nm. As mentioned before, the energy splitting between the two modes is due to the different interaction with the underlying silicon substrate. The P-mode, which is a plasmonic excitation normal to the surface substrate, presents strong interaction with the underneath substrate. Since silicon refractive index is high in the visible spectral range, n ≃ 4, a huge red-shift of its plasmon energy is observed. Conversely the S-mode, which is a plasmonic excitation parallel to the surface, localizes the charge further away from the substrate, resulting in a much weaker interaction than that for the P-mode. Moreover, depending on the polarization selected we can enhance or reduce one resonance peak in relation to the other one^51^,^52^.

In comparison to single nanoparticles, dimer nanoparticle spectra have a more complex behavior^53^ (Fig. 3c). The loss of azimuthal symmetry in dimer nanostructures produces the splitting of two different modes oscillating parallel to the surface substrate; the 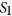 mode is a plasmonic excitation occurring along the dimer short axis, while the 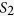 mode is a dipolar excitation acting on the long dimer axis (see right bottom insets of Fig. 3c). While the 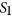 mode has a resonant energy similar to the 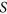 mode of the single nanoparticle (note the dimer short axis is equal to the nanoparticle diameter), the 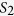 mode presents a remarkable red-shift (around 580 nm) because the plasmonic oscillation occurs along the major axis of the dimer. The experimentally observed spectral position of the plasmonic modes of single and dimer nanoparticles is in good agreement with both analytical theory and numerical simulations (see Supplementary Information Supp. 1 for more technical details). The results are not contradictory to previous works that study single and dimer nanoparticles onto low refractive index glass substrates^54^,^55^. Moreover, as the dimer P mode is a normally-oriented plasmonic excitation occurring along a short dimer (equal to the nanoparticle diameter), its resonant energy is similar to that of the P mode of the single nanoparticle.

It is important to remark that all the detailed spectral features here reported for individual single and dimer nanoparticles are readily available also for all of the nanoparticles present in the sample (Fig. 2a) due to the high throughput of the SMMS technique that allows the spectral characterization of large sample areas in few minutes.

### Analysis of the scattering emission shape of plasmonic nanoparticles

**Figure 4:**
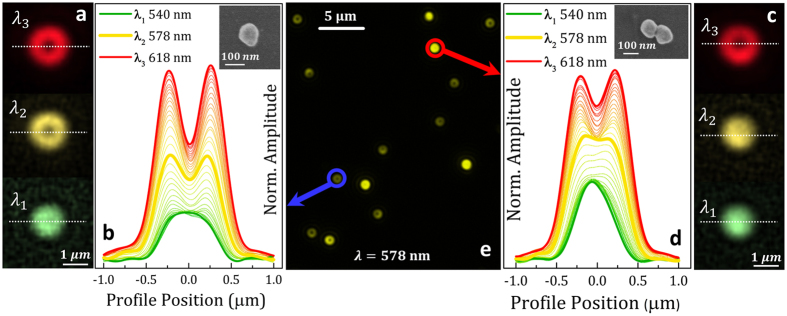
Spatial Mapping of the scattering emission of single and dimer nanoparticles. (**a**) Normalized dark-field images of the same single nanoparticle characterized in Fig. 3b, measured at three different wavelengths: λ_1_ = 540 nm, λ_2_ = 578 nm, λ_3_ = 618 nm. Images have been obtained with P-polarized illumination. (**b**) Spatial profile of the wavelength-dependent scattering emission of the same single nanoparticle shown in Figs 3b and 4a. The spatial profiles obtained with P-polarized light were measured along the white dotted lines drawn in (**a**); the light wavelength was changed from 540 nm to 618 nm with steps of 2 nm. The inset is a SEM image of the single nanoparticle under analysis. (**c**) Normalized dark-field images of the dimer nanoparticle previously characterized in Fig. 3c, obtained at three different wavelengths: λ_1_ = 540 nm, λ_2_ = 578 nm, λ_3_ = 618 nm. Images were obtained with P-polarized illumination. (**d**) Spatial profile of the wavelength-dependent scattering emission of the same dimer nanoparticle shown in Fig. 3c and 4c. The spatial profiles obtained with P-polarized light were measured along the white dotted lines drawn in (**c**); the light wavelength was changed from 540 nm to 618 nm with steps of 2 nm. The inset is a SEM image of the dimer nanoparticle under analysis. (**e**) Optical dark-field image of single and dimer nanoparticles obtained by illuminating the sample surface at λ_2_ = 578 nm and P-polarized light; the blue circle identifies the single nanoparticle of (**a,b**), while the red circle corresponds to the dimer shown in (**c,d**).

The high spatial resolution of the SMMS technique allows also the detailed spatial analysis of the scattering emission of individual plasmonic nanoparticles. The shape of the far-field scattering emission is an important fingerprint both for the identification of different plasmonic modes in the same nanoparticle and for the study of nanoparticle to substrate plasmonic coupling. Theory predicts the characteristic spatial distribution of the far-field emission depending on the plasmonic modes considered^20^,^32^,^56^. Since the size of the nanoparticles under study is much smaller than the wavelength of the incident light, the plasmon resonances of single and dimer nanoparticles can be treated as polarizable electric point dipoles. The dipole moment emanating parallel to the substrate surface (S-mode) generates a solid bright far-field scattering pattern, while a dipole moment oscillating normal to the substrate (P-mode) relates to a characteristic doughnut shape. Single gold nanoparticles here under study have a clear spectral separation between the S-mode and the P-mode due to the different coupling with the underneath silicon substrate and, for this reason, we expect to observe two distinct scattering patterns in the same nanoparticle depending on the wavelength selected. In the following we demonstrate the capability of the SMMS technique to experimentally analyze the scattering emission shapes in single and dimer nanoparticles at different wavelengths of the excitation light. Dark-field images in Fig. 4a are the far-field scattering patterns of the same single nanoparticle analyzed in Fig. 3b, obtained at three different wavelengths of the excitation light: at the maximum of the 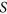 mode (green pattern, λ_1_ = 540 nm), at the maximum of the 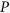 mode (red pattern, λ_3_ = 618 nm) and at an intermediate wavelength (yellow pattern, λ_2_ = 578 nm). This single nanoparticle has a solid bright scattering at the maximum of the S-mode (λ_1_, green solid pattern) and a typical doughnut-shape at the maximum of the P-mode (λ_3_, red doughnut pattern). At the intermediate wavelength (λ_2_ = 578 nm) the scattering still has a doughnut-shape emission (yellow doughnut pattern), because the scattering is dominated by the 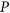 mode. The entire evolution from spherical to doughnut-shape emission can be followed by the SMMS technique with high spatial resolution, as shown in Fig. 4b, where the wavelength-dependence of the spatial profile of a single nanoparticle is plotted (spatial profile at the position marked by the white dotted lines in Fig. 4a). The unique capability of the SMMS technique to quantitatively characterize the spatial profile evidences that the doughnut shaped scattering emission is significantly broader than the solid bright scattering.

Scattering patterns were also analyzed for the dimer nanoparticle previously characterized in Fig. 3c. Dark-field images of the dimer (see Fig. 4c) obtained both at the maximum of the S-mode (λ_1_) and of the P-mode (λ_3_) are qualitatively similar to the case of the single nanoparticle, while a notable difference can be observed at the intermediate wavelength (yellow, λ_2_ = 578 nm). At this wavelength, the dimer scattering emission is dominated by the 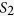 mode which produces a solid bright scattering pattern. The entire wavelength-dependence of the dimer spatial profiles shown in Fig. 4d allows appreciating the slower transition from spherical to doughnut shape due to the dominancy of the 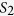 mode at 578 nm. A video showing the cited transitions in the scattering profiles of a single and a dimer nanoparticle can be found in Supplementary Information (Supp. 3).

As single and dimer nanoparticles exhibit different scattering shapes at some selected wavelengths, the SMMS technique offers a straightforward method to easily distinguish single and dimer nanoparticles from a one shot image. In our experimental conditions, single and dimer nanoparticles can be easily discerned by illuminating the sample surface at the intermediate wavelength λ_2_ = 578 nm (see Fig. 4e); single nanoparticles present a doughnut-shaped scattering emission, while dimers emit with a solid bright pattern. This analysis is possible thanks to two distinctive characteristics of the SMMs technique; its high spatial resolution of 40 nm at magnification of 100× and the capability to select the wavelength of the excitation light. It is important to remark that this experimental analysis is not feasible with a standard dark-field micro-spectroscopy experimental setup, due to the low spatial resolution and the need of image stitching that further deteriorates the shape of the scattering emission.

More importantly, the high signal-to-noise ratio measurements proved by the SMMS technique make now possible to observe previously undetectable scattering features coming from individual nanoparticles; for example, at the excitation wavelength of 540 nm, the scattering pattern of the single nanoparticle is significantly broader than that of the dimer (Fig. 4b,d). Thus, the SMMS provides a new avenue for the spatially-resolved characterization of the far field scattering emission of single and coupled plasmonic systems that is key for the design of nanoantennas^25^, the development of plasmonic rulers^20^ and to correct variations in SERS enhancement^57^,^58^,^59^ from nanostructure to nanostructure. Although we have focused our study in single and dimer nanoparticles, the SMMS technique has the potential to properly distinguish also trimers, tetramers and larger agglomerates. However, for such analysis, a CCD camera with an extended working spectral range should be used.

### Experimental Characterization of the diameter of nanoparticles

**Figure 5:**
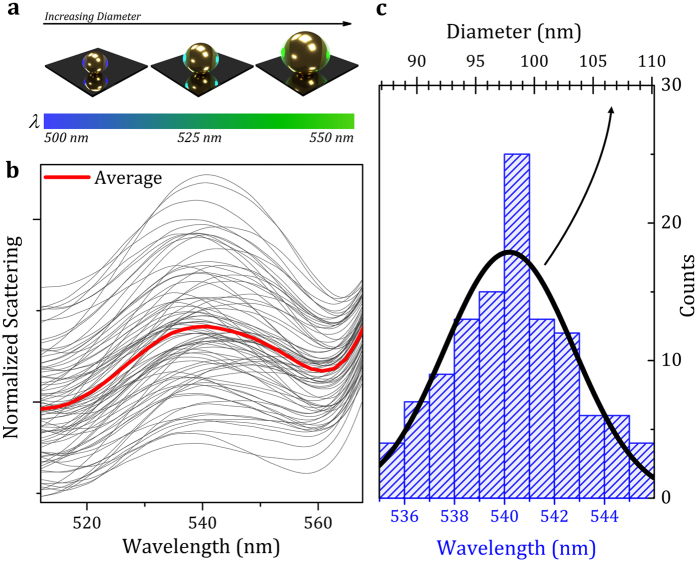
Measurement of the Diameter of Single Nanoparticles. (**a**) Schematic drawing representing the wavelength-dependence of the 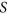 mode on the nanoparticle size. (**b**) Normalized S-polarized spectra of the 177 single nanoparticles in the spectral range near to the 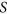 mode; red solid line represents the average of all the scattering spectra. (**c**) Histogram distribution of the spectral position of the 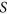 mode (bottom axis). Solid black line represents the calculated Gaussian distribution of nanoparticle diameter population (top axis).

We finally challenge the technique with a practical application; the characterization of the diameter of hundreds of single nanoparticles. To this aim, we have first identified and eliminated from the analysis all the dimers and only the remaining 177 single nanoparticles have been analyzed. This process was automatically performed with a custom-built routine in just few seconds. The nanoparticles appear of different colors in the dark-field images due to the differences in their diameter when there are no changes of the surrounding medium (Fig. 5a). As the S-mode, which is parallelly oriented to the substrate surface, is poorly influenced by the underneath substrate, it will be assumed hereinafter that the spectral shift of its plasmonic mode depends almost exclusively on differences of the nanoparticle size.

The S-mode spectra of all the 177 single nanoparticles (dark gray solid lines) and their average value (red solid line) are shown in Fig. 5b; these experimental results were obtained with S-polarized light. The maximum of the 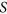 mode spectral position of single nanoparticles presents small spectral changes around the average value of ≃540 nm. We therefore calculated the histogram distribution of the maximum of the S-mode (Fig. 5c) by fitting all the nanoparticle spectra with two Lorentzian curves that take into account both the S-mode and the P-mode; the calculus of the histogram distribution was obtained by including all the nanoparticle spectra with a fit goodness higher than 0.99. The relation between the spectral position of the 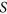 mode and the nanoparticle diameter was therefore calculated^58^,^59^ by considering the analytical theory for coated spheres. We achieve an accuracy of 1 nm in the determination of the nanoparticle diameter. This accuracy is given by the spectral accuracy of our experimental setup and the goodness of the fitting procedure. More technical details can be found in Supplementary Information Supp. 2. The Gaussian distribution of the nanoparticle diameter shown in Fig. 5c estimates an average nanoparticle diameter of 98 nm ± 5.4 nm that is in good agreement with the nominal value and uncertainty specified by the manufacturer (nominal diameter of 100 nm with an uncertainty of 4%). The full characterization and analysis of the 177 nanoparticles were completed in 2.5 minutes. Advantageously, the diameter for each individual nanoparticle can be easily assigned to the particular scatterer because with the SMMS the spatial position of each individual nanoparticle is well-known. This aspect results particularly useful for the development of sensing devices with large multiplexing capability.

## Conclusions

The technique presented here, Spatially Multiplexed MicroSpectrophotometry (SMMS) is capable of performing polarization-resolved dark-field spectral analysis of hundreds of nanoparticles with single particle resolution in minutes. The technique allows also accurate characterization of the spatial distribution of the scattered light (scattering patterns) with a spatial resolution of 40 nm. The potential of this new and versatile technique is demonstrated here through dark field spectral characterization of 241 single and dimer plasmonic nanoparticles. The tuning of the excitation light wavelength has allowed us to separately characterize the scattering profiles related to the S-mode and the P-mode on the same nanoparticle. The possibility to finely tune the scattering emission of a nanoparticle by changing the illumination wavelength and the SMMS capability to quantitatively and accurately characterize the scattering profile opens up new routes for the design and characterization of plasmonic devices. The SMMS is optimally suited for characterizing a variety of nanoscale scatterers of different size, shape and material, as well as large assemblies of plasmonic nanoparticles, not amenable to techniques that can only address small surface areas. Thus, fast and high resolution spatially resolved spectral characterization of large sample surfaces is now feasible, offering a new experimental tool in a multitude of application fields in nanoplasmonics. We have implemented here the technique in reflection mode, but the approach could also be implemented in extinction configurations with the same cited advantages. The study of nanoparticle to substrate plasmonic coupling can also be performed in extinction configuration by using thin films of high refractive index materials deposited on a transparent substrate.

## Methods

### Experimental Setup

The practical realization of the instrument was implemented by using a Xenon lamp and a VI-IR motorized monochromator (Tunable PowerArc™ Illuminator, Optical Build Blocks) coupled via a liquid light guide (LLG0538-6, Thorlabs), a linear polarizer (Prinz, M42) and a collimating adapter (LLG5A5-A, Thorlabs) to a commercial optical microscope column (Nikon Eclipse). In order to avoid the overlapping of the second-order diffraction of light coming from the monochromator, a long-wave pass optical filter (GG475, Microbeam) was placed along the detection arm of the experimental setup. Measurements were acquired with a Peltier-cooled color CCD camera (DS-Ri1, Nikon, working spectral range from 400 nm to 700 nm) connected to a PC through a camera controller (DS-U3, Nikon).

### Nanoparticle Preparation

A silicon wafer was cut in pieces of 0.5 × 0.5 mm, cleaned with piranha solution (H_2_SO_4_:H_2_O_2_, 2:1) for 5 minutes, rinsed three times with Milli-Q^®^ water and dried under a stream of N_2_. The silicon surfaces were dipped into a solution 0.001% w/v of poly-lisyne (Sigma-Aldrich^®^) in Milli-Q^®^ water for 1 hour and 25 °C under gentle agitation. The samples were washed twice with Milli-Q^®^ water and dried with N_2_. Right after, the surfaces were dipped into a solution 1 × 10^8 ^nps/mL of 100 nm diameter gold nanoparticles (Nanopartz™, USA) in Milli-Q^®^ water for 1 hour and 25 °C under agitation. The silicon surfaces were removed from the gold nanoparticles solution, rinsed vigorously with Milli-Q^®^ water and dried under a stream of N_2_.

### Raw Data Normalization and Data Analysis

Each image frame containing a wavelength-component of the sample scattering was corrected for the weak scattering coming from the substrate by subtracting pixel by pixel the silicon background scattering. Then, it was normalized for the wavelength response of the apparatus by dividing pixel by pixel by the scattering spectrum obtained from a white scattering standard. A custom-built routine developed in Matlab^®^ individuates the spatial coordinates of every nanoparticle and it ensures an accurate spectral and spatial analysis of all the individual nanoparticles. The algorithm developed allows also the elimination of saturated scatterers produced by the presence of large agglomerates, debris or groups of individual nanoparticles placed at distances smaller than the diffraction limit.

## Additional Information

**How to cite this article**: Pini, V. *et al.* Spatially multiplexed dark-field microspectrophotometry for nanoplasmonics. *Sci. Rep.* **6**, 22836; doi: 10.1038/srep22836 (2016).

## Supplementary Material

Supplementary Movie

Supplementary Information
