# Supplementary material for: Spatially multiplexed dark-field microspectrophotometry for nanoplasmonics
Source: Sci Rep. 2016 Mar 8;6:22836. doi: 10.1038/srep22836 (PMC4782137; doi:10.1038/srep22836)
Supplement: Supplementary Information [file srep22836-s2.pdf]

SUPPLEMENTARY INFORMATION

Spatially multiplexed dark-field microspectrophotometry for nanoplasmonics

V. Pini, P.M. Kosaka, J.J. Ruz, O. Malvar, M. Encinar, J. Tamayo* and M. Calleja*

*Corresponding author: jtamayo@imm.cnm.csic.es, mcalleja@imm.cnm.csic.es

Supp1. Theoretical Characterization of Single and Dimer Nanoparticles

When a gold nanoparticle is bound to a substrate surface, the degeneration of the plasmon modes parallel and perpendicular to the substrate is broken. Thus the presence of two resonance peaks in single nanoparticle spectra can be associated to dipolar plasmonic oscillations parallel and normal to the surface of the silicon substrate. A qualitative interpretation can be obtained from the analytical Mie scattering theory[1] for coated spheres; scattering spectra shown in Fig. Supp1 were obtained for different refractive indexes of the surrounding environment. We assume a gold nanoparticle diameter of 100 nm and a polymer layer 5 nm thick, compatible with the specifications of the manufacturer (Nanopartz, USA). The wavelength-dependence of the gold optical properties was considered in the calculation by using the data tabulated by Johnson[2], whereas the refractive index of the polymer layer was fixed at 1.5. The high energy peak, named in the main text as S mode, is dominated by the refractive index of the air environment and its spectral position resembles the green curve in Fig. Supp1, calculated for a refractive index n = 1. Instead the low energy peak, named in the main text as P mode, will be more influenced by the higher refractive index of the silicon substrate and its spectral position will be shifted toward the red curve represented in Fig. Supp1, calculated for a refractive index n = 1.6.

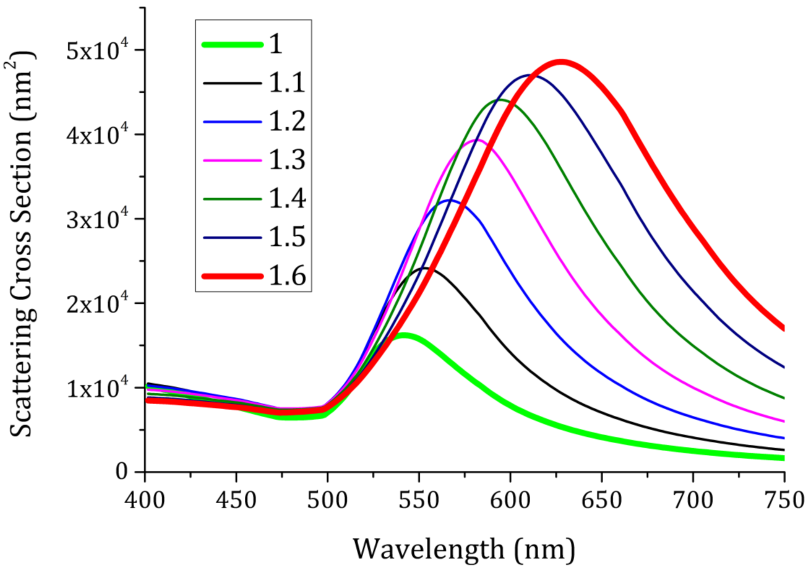

Figure Supp1. Theoretical scattering cross section of a 100 nm gold nanoparticle coated with a 5 nm thick polymer layer. Calculation was performed for different environment refractive indexes (n = 1, 1.1, 1.2, 1.3, 1.4, 1.5, and 1.6).

In order to corroborate our hypothesis, a numerical simulation of a coated gold nanoparticle attached to a silicon substrate and illuminated using a high incidence angle was performed (Fig. Supp2). The scattering spectrum obtained by the numerical simulation is in good agreement to the experimental results. The two insets in Fig. Supp2 are the square of the intensity of the electromagnetic field of the two dipole peaks.

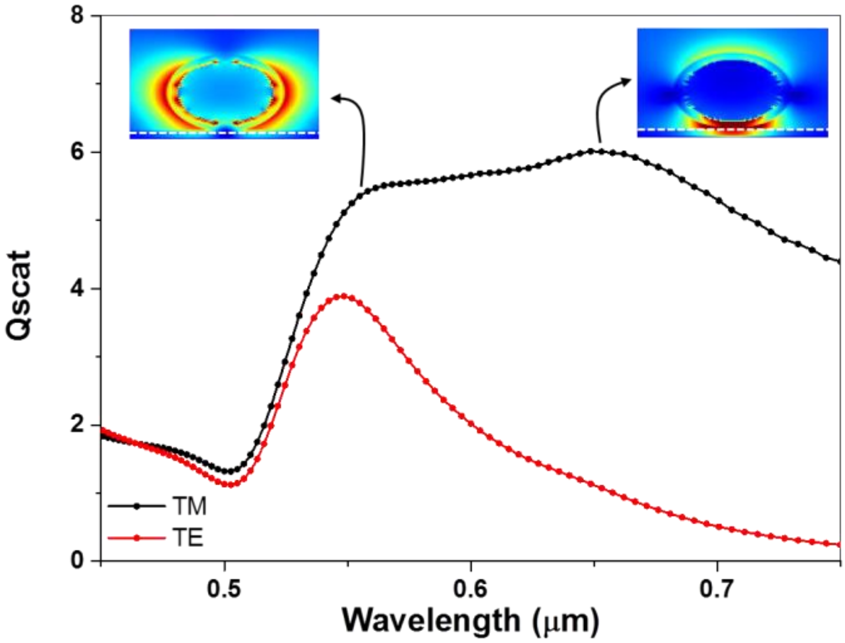

Figure Supp2. Numerical simulation of the normalized scattering cross section of a polymer coated gold nanoparticle attached to a silicon substrate illuminated with transversal magnetic plane wave (TM or P-polarization) and transversal electric plane wave (TE or S-polarization). Numerical analysis was performed by setting an incident angle of ~ 40 degrees. The insets are the models of the electromagnetic field of the two plasmon resonances; the white dashed lines represent the silicon surface.

Dimer nanoparticle spectra present a more complex behavior than single nanopoarticles. In addition to the previously described peaks, dimer nanoparticles have a third peak, named in the main text as S_2 mode that corresponds to a dipole oscillation along the longitudinal axis of the dimer. Since this dipole mode oscillates parallel to the substrate surface, the influence of the underneath substrate is negligible and the dimer can be treated with a semi-analytical model described by Garcia de Abajo[3]. In Fig. Supp3 we reported the normalized spectra of a dimer nanoparticle for different inter-particle distances; light illumination is polarized along the longitudinal axis of the dimer. Theoretical spectra confirmed a dipole plasmon mode S_2 around 580 nm for inter-particle distances about 5-10 nm.

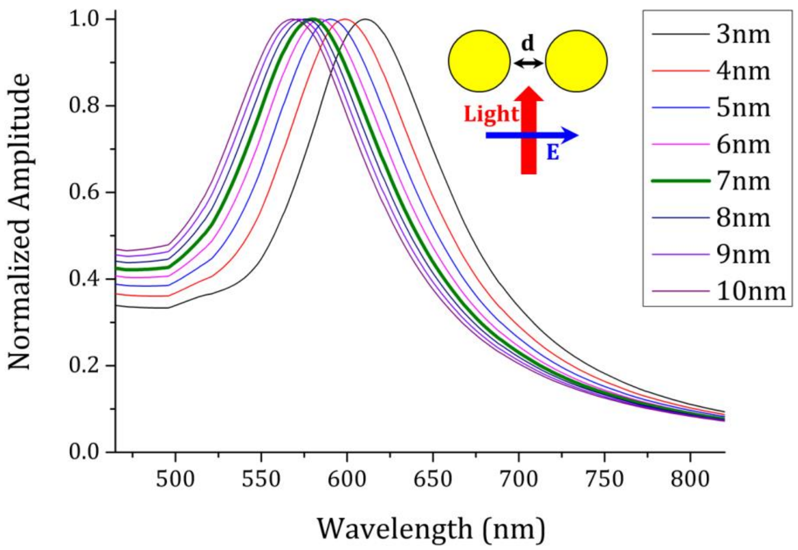

Figure Supp3. Normalized spectra of a dimer for different inter-particle distances (d in the inset); light illumination is polarized along the longitudinal axis of the dimer. The inset in the figure is a schematic drawing of a dimer. Calculations were performed by including the wavelength-dependence of gold optical properties tabulated in Reference[2] and by fixing the refractive index of the surrounding environment to 1.

Supp2. Calculation of Single Particle Diameter

As described in the main text, the spectral position of the $\boldsymbol{S}$ mode allows evaluating the size of the single nanoparticle under study. In fact, the S-mode has a negligible influence of the underneath substrate and thus, the spectral position of the maximum of the S-mode can be determined from the analytical Mie scattering theory for coated spheres[1]. For the calculation we considered coated gold nanoparticle spheres with a polymer coating layer of 5 nm given by the manufacturer specifications. Calculations were performed by including the wavelength-dependence of the gold optical properties[2], while the refractive index of the polymer layer was fixed to 1.5. For each nanoparticle diameter $\boldsymbol{D}$ we calculated the spectral position of the maximum of the scattering resonance peak $\boldsymbol{S}$**,** obtaining the following linear relationship

$$\boldsymbol{S} = \boldsymbol{\alpha} + \boldsymbol{\beta}\,\boldsymbol{D}, \qquad \text{(S.1)}$$

where $\alpha \cong 494.5$ and $\beta \cong 0.467$; both S and D in equation (S.1) are expressed in nm. Thus, the nanoparticle diameter can be obtained by simply inverting the equation (S.1).

Supp3. Spatial scattering mapping of a single and a dimer nanoparticle

A video showing the spatial scattering emission of a single (left) and dimer (right) nanoparticle illuminated at different wavelengths (from 510 nm to 630 nm) that illustrates the transition from the solid bright pattern to the doughnut-shaped scattering emission.

Supplementary references

1 Bohren, C. F. & Huffman, D. R. *Absorption and scattering of light by small particles*. (John Wiley & Sons, 2008).

2 Johnson, P. B. & Christy, R. W. Optical Constants of the Noble Metals. *Phys. Rev. B* **6**, 4370-4379 (1972).

3 García de Abajo, F. J. Multiple scattering of radiation in clusters of dielectrics. *Phys. Rev. B* **60**, 6086-6102 (1999).
